# LB2306. Population Pharmacokinetic (PPK), Pharmacokinetic/Pharmacodynamic attainment (PTA), and Clinical Pharmacokinetic/Pharmacodynamic (PK/PD) Analyses for Sulbactam-Durlobactam (SUL-DUR) to Support Dose Selection for the Treatment of *Acinetobacter baumannii-calcoaceticus* Complex (ABC) Infections

**DOI:** 10.1093/ofid/ofac492.1896

**Published:** 2022-12-15

**Authors:** Sujata M Bhavnani, Sujata M Bhavnani, Sujata M Bhavnani, Christopher M Rubino, Jeffrey P Hammel, Anthony P Cammarata, Kajal Larson, Kathryn Liolios, Sarah McLeod, Alita Miller, Paul G Ambrose, Ruben Tommasi, John O'Donnell

**Affiliations:** Institute for Clinical Pharmacodynamics, Schenectady, NY; Institute for Clinical Pharmacodynamics, Schenectady, NY; Institute for Clinical Pharmacodynamics, Schenectady, NY; Institute for Clinical Pharmacodynamics, Schenectady, NY; Institute for Clinical Pharmacodynamics, Schenectady, NY; Institute for Clinical Pharmacodynamics, Schenectady, NY; Entasis Therapeutics, Waltham, Massachusetts; Institute for Clinical Pharmacodynamics, Schenectady, NY; Entasis Therapeutics, Waltham, Massachusetts; Entasis Therapeutics, Waltham, Massachusetts; Institute for Clinical Pharmacodynamics, Schenectady, NY; Entasis Therapeutics, Waltham, Massachusetts; Entasis Therapeutics, Waltham, Massachusetts

## Abstract

**Background:**

SUL-DUR is a β-lactam/β-lactamase inhibitor combination in development for the treatment of ABC infections, which are often severe and associated with substantial mortality. PPK, PTA, and clinical PK/PD analyses were conducted using all available PK and efficacy data to support SUL-DUR dose selection.

**Methods:**

PPK analyses were performed using PK data from 373 subjects, including 110 patients who received SUL-DUR and underwent PK sampling in the pivotal Phase 3 (ATTACK) trial. SUL-DUR concentrations in epithelial lining fluid (ELF) from healthy subjects were utilized to predict SUL-DUR ELF concentrations in Phase 3 patients. Covariate analyses evaluated the impact of demographic factors on PK exposures. The PPK model, non-clinical PK/PD data, and in vitro surveillance data were used to assess the PTA by MIC in simulated patients with ABC infections. Clinical PK/PD relationships were assessed using dichotomous and time-to-event efficacy endpoints from the ATTACK trial.

**Results:**

Two-compartment models with linear kinetics best characterized SUL and DUR PK. Consistent with high renal clearance of SUL-DUR, dose adjustments are needed in patients with renal impairment or augmented renal function. Body weight, site of infection, and East Asian region (which grouped patients from mainland China, Taiwan, and South Korea vs others) were statistically significant covariates in the PPK analysis but were not clinically relevant. No other statistical or clinically relevant covariates were identified. As shown in Figures A and B for plasma and ELF, respectively, the PTA was ≥ 90% for pathogens with an MIC ≤ 4 µg/mL across renal function categories in simulated patients, using PK/PD targets associated with a 1-log kill_10_. No relationships were observed between PK/PD indices and efficacy endpoints, consistent with most patients achieving SUL and DUR exposures above nonclinical PK/PD targets for efficacy.

Percent PTA by MIC on Day 1 based on the assessment of sulbactam %T>MIC ≥ 50% and durlobactam AUC:MIC ratio ≥ 10 targets and free-drug plasma (A) and total-drug ELF (B) exposures among simulated patients by CLcr group after administration of sulbactam 1 g/ durlobactam 1 g IV q6h and dosing regimens adjusted for renal impairment and augmented renal function

**Figure:**
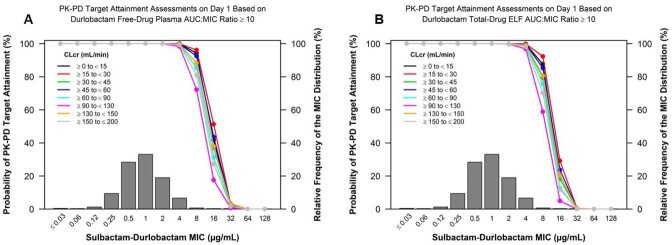

**Conclusion:**

The simulated plasma and ELF exposures yielded a high PTA, which when combined with the favorable efficacy and safety findings from ATTACK (abstract #1266378), support a dose of 1.0 g sulbactam/1.0 g durlobactam via a 3-hour infusion, every 6 hours in patients with normal renal function and renal function-based dose adjustments.

**Disclosures:**

**Sujata M. Bhavnani, PharmD; MS; FIDSA**, Adagio Therapeutics, Inc.: Grant/Research Support|Amplyx Pharmaceuticals, Inc.: Grant/Research Support|AN2 Therapeutics: Grant/Research Support|Antabio SAS: Grant/Research Support|Arcutis Biotherapeutics, Inc.: Grant/Research Support|B. Braun Medical Inc.: Grant/Research Support|Basilea Pharmaceutica: Grant/Research Support|Boston Pharmaceuticals: Grant/Research Support|Bravos Biosciences: Ownership Interest|Celdara Medical LLC: Grant/Research Support|Cidara Therapeutics Inc.: Grant/Research Support|Cipla USA: Grant/Research Support|Crestone Inc.: Grant/Research Support|CXC: Grant/Research Support|Debiopharm International SA: Grant/Research Support|Entasis Therapeutics: Grant/Research Support|Evopoint Biosciences Co.: Grant/Research Support|Fedora Pharmaceuticals: Grant/Research Support|GlaxoSmithKline: Grant/Research Support|Hoffmann-La Roche: Grant/Research Support|ICPD: Ownership Interest|ICPD Biosciences, LLC.: Ownership Interest|ICPD Holdings: Ownership Interest|ICPD Technologies: Ownership Interest|Insmed Inc.: Grant/Research Support|Iterum Therapeutics Limited: Grant/Research Support|Kaizen Bioscience, Co.: Grant/Research Support|KBP Biosciences USA: Grant/Research Support|Lassen Therapeutics: Grant/Research Support|Matinas Biopharma: Grant/Research Support|Meiji Seika Pharma Co., Ltd.: Grant/Research Support|Menarini Ricerche S.p.A.: Grant/Research Support|Mutabilis: Grant/Research Support|Nabriva Therapeutics AG: Grant/Research Support|Novartis Pharmaceuticals Corp.: Grant/Research Support|Paratek Pharmaceuticals, Inc.: Grant/Research Support|PureTech Health: Grant/Research Support|Sfunga Therapeutics: Grant/Research Support|Spero Therapeutics: Grant/Research Support|Suzhou Sinovent Pharmaceuticals Co.: Grant/Research Support|TauRx Therapeutics: Grant/Research Support|Tetraphase Pharmaceuticals: Grant/Research Support|tranScrip Partners: Grant/Research Support|Utility Therapeutics: Grant/Research Support|Valanbio Therapeutics, Inc.: Grant/Research Support|VenatoRx: Grant/Research Support|Wockhardt Bio AG: Grant/Research Support **Sujata M. Bhavnani, PharmD; MS; FIDSA**, Adagio Therapeutics, Inc.: Grant/Research Support|Amplyx Pharmaceuticals, Inc.: Grant/Research Support|AN2 Therapeutics: Grant/Research Support|Antabio SAS: Grant/Research Support|Arcutis Biotherapeutics, Inc.: Grant/Research Support|B. Braun Medical Inc.: Grant/Research Support|Basilea Pharmaceutica: Grant/Research Support|Boston Pharmaceuticals: Grant/Research Support|Bravos Biosciences: Ownership Interest|Celdara Medical LLC: Grant/Research Support|Cidara Therapeutics Inc.: Grant/Research Support|Cipla USA: Grant/Research Support|Crestone Inc.: Grant/Research Support|CXC: Grant/Research Support|Debiopharm International SA: Grant/Research Support|Entasis Therapeutics: Grant/Research Support|Evopoint Biosciences Co.: Grant/Research Support|Fedora Pharmaceuticals: Grant/Research Support|GlaxoSmithKline: Grant/Research Support|Hoffmann-La Roche: Grant/Research Support|ICPD: Ownership Interest|ICPD Biosciences, LLC.: Ownership Interest|ICPD Holdings: Ownership Interest|ICPD Technologies: Ownership Interest|Insmed Inc.: Grant/Research Support|Iterum Therapeutics Limited: Grant/Research Support|Kaizen Bioscience, Co.: Grant/Research Support|KBP Biosciences USA: Grant/Research Support|Lassen Therapeutics: Grant/Research Support|Matinas Biopharma: Grant/Research Support|Meiji Seika Pharma Co., Ltd.: Grant/Research Support|Menarini Ricerche S.p.A.: Grant/Research Support|Mutabilis: Grant/Research Support|Nabriva Therapeutics AG: Grant/Research Support|Novartis Pharmaceuticals Corp.: Grant/Research Support|Paratek Pharmaceuticals, Inc.: Grant/Research Support|PureTech Health: Grant/Research Support|Sfunga Therapeutics: Grant/Research Support|Spero Therapeutics: Grant/Research Support|Suzhou Sinovent Pharmaceuticals Co.: Grant/Research Support|TauRx Therapeutics: Grant/Research Support|Tetraphase Pharmaceuticals: Grant/Research Support|tranScrip Partners: Grant/Research Support|Utility Therapeutics: Grant/Research Support|Valanbio Therapeutics, Inc.: Grant/Research Support|VenatoRx: Grant/Research Support|Wockhardt Bio AG: Grant/Research Support **Sujata M. Bhavnani, PharmD; MS; FIDSA**, Adagio Therapeutics, Inc.: Grant/Research Support|Amplyx Pharmaceuticals, Inc.: Grant/Research Support|AN2 Therapeutics: Grant/Research Support|Antabio SAS: Grant/Research Support|Arcutis Biotherapeutics, Inc.: Grant/Research Support|B. Braun Medical Inc.: Grant/Research Support|Basilea Pharmaceutica: Grant/Research Support|Boston Pharmaceuticals: Grant/Research Support|Bravos Biosciences: Ownership Interest|Celdara Medical LLC: Grant/Research Support|Cidara Therapeutics Inc.: Grant/Research Support|Cipla USA: Grant/Research Support|Crestone Inc.: Grant/Research Support|CXC: Grant/Research Support|Debiopharm International SA: Grant/Research Support|Entasis Therapeutics: Grant/Research Support|Evopoint Biosciences Co.: Grant/Research Support|Fedora Pharmaceuticals: Grant/Research Support|GlaxoSmithKline: Grant/Research Support|Hoffmann-La Roche: Grant/Research Support|ICPD: Ownership Interest|ICPD Biosciences, LLC.: Ownership Interest|ICPD Holdings: Ownership Interest|ICPD Technologies: Ownership Interest|Insmed Inc.: Grant/Research Support|Iterum Therapeutics Limited: Grant/Research Support|Kaizen Bioscience, Co.: Grant/Research Support|KBP Biosciences USA: Grant/Research Support|Lassen Therapeutics: Grant/Research Support|Matinas Biopharma: Grant/Research Support|Meiji Seika Pharma Co., Ltd.: Grant/Research Support|Menarini Ricerche S.p.A.: Grant/Research Support|Mutabilis: Grant/Research Support|Nabriva Therapeutics AG: Grant/Research Support|Novartis Pharmaceuticals Corp.: Grant/Research Support|Paratek Pharmaceuticals, Inc.: Grant/Research Support|PureTech Health: Grant/Research Support|Sfunga Therapeutics: Grant/Research Support|Spero Therapeutics: Grant/Research Support|Suzhou Sinovent Pharmaceuticals Co.: Grant/Research Support|TauRx Therapeutics: Grant/Research Support|Tetraphase Pharmaceuticals: Grant/Research Support|tranScrip Partners: Grant/Research Support|Utility Therapeutics: Grant/Research Support|Valanbio Therapeutics, Inc.: Grant/Research Support|VenatoRx: Grant/Research Support|Wockhardt Bio AG: Grant/Research Support **Christopher M. Rubino, PharmD**, Adagio Therapeutics: Grant/Research Support|Amplyx Pharmaceuticals, Inc.: Grant/Research Support|AN2 Therapeutics: Grant/Research Support|Antabio SAS: Grant/Research Support|Arcutis Biotherapeutics, Inc.: Grant/Research Support|B. Braun Medical Inc.: Grant/Research Support|Basilea Pharmaceutica: Grant/Research Support|Boston Pharmaceuticals: Grant/Research Support|Bravos Biosciences: Ownership Interest|Celdara Medical LLC: Grant/Research Support|Cidara Therapeutics Inc.: Grant/Research Support|Cipla USA: Grant/Research Support|Crestone Inc.: Grant/Research Support|CXC: Grant/Research Support|Debiopharm International SA: Grant/Research Support|Entasis Therapeutics: Grant/Research Support|Evopoint Biosciences Co.: Grant/Research Support|Fedora Pharmaceuticals: Grant/Research Support|GlaxoSmithKline: Grant/Research Support|Hoffmann-La Roche: Grant/Research Support|ICPD: Ownership Interest|ICPD Biosciences, LLC.: Ownership Interest|ICPD Holdings: Ownership Interest|ICPD Technologies: Ownership Interest|Insmed Inc.: Grant/Research Support|Iterum Therapeutics Limited: Grant/Research Support|Kaizen Bioscience, Co.: Grant/Research Support|KBP Biosciences USA: Grant/Research Support|Lassen Therapeutics: Grant/Research Support|Matinas Biopharma: Grant/Research Support|Meiji Seika Pharma Co., Ltd.: Grant/Research Support|Melinta Therapeutics: Grant/Research Support|Menarini Ricerche S.p.A.: Grant/Research Support|Mutabilis: Grant/Research Support|Nabriva Therapeutics AG: Grant/Research Support|Novartis Pharmaceuticals Corp: Grant/Research Support|Paratek Pharmaceuticals, Inc.: Grant/Research Support|PureTech Health: Grant/Research Support|Sfunga Therapeutics: Grant/Research Support|Spero Therapeutics: Grant/Research Support|Suzhou Sinovent Pharmaceuticals Co.: Grant/Research Support|TauRx Therapeutics: Grant/Research Support|Tetraphase Pharmaceuticals: Grant/Research Support|tranScrip Partners: Grant/Research Support|Utility Therapeutics: Grant/Research Support|Valanbio Therapeutics, Inc.: Grant/Research Support|VenatoRx: Grant/Research Support|Wockhardt Bio AG: Grant/Research Support **Jeffrey P. Hammel, MS**, Adagio Therapeutics, Inc.: Grant/Research Support|Amplyx Pharmaceuticals, Inc.: Grant/Research Support|AN2 Therapeutics: Grant/Research Support|Antabio SAS: Grant/Research Support|Arcutis Biotherapeutics, Inc.: Grant/Research Support|B. Braun Medical Inc.: Grant/Research Support|Basilea Pharmaceutica: Grant/Research Support|Boston Pharmaceuticals: Grant/Research Support|Celdara Medical LLC: Grant/Research Support|Cidara Therapeutics Inc.: Grant/Research Support|Cipla USA: Grant/Research Support|Crestone Inc.: Grant/Research Support|CXC: Grant/Research Support|Debiopharm International SA: Grant/Research Support|Entasis Therapeutics: Grant/Research Support|Evopoint Biosciences Co.: Grant/Research Support|Fedora Pharmaceuticals: Grant/Research Support|GlaxoSmithKline: Grant/Research Support|Hoffmann-La Roche: Grant/Research Support|Insmed Inc.: Grant/Research Support|Iterum Therapeutics Limited: Grant/Research Support|Kaizen Bioscience, Co.: Grant/Research Support|KBP Biosciences USA: Grant/Research Support|Lassen Therapeutics: Grant/Research Support|Matinas Biopharma: Grant/Research Support|Meiji Seika Pharma Co., Ltd.: Grant/Research Support|Melinta Therapeutics: Grant/Research Support|Menarini Ricerche S.p.A.: Grant/Research Support|Mutabilis: Grant/Research Support|Nabriva Therapeutics AG: Grant/Research Support|Novartis Pharmaceuticals Corp.: Grant/Research Support|Paratek Pharmaceuticals, Inc.: Grant/Research Support|PureTech Health: Grant/Research Support|Sfunga Therapeutics: Grant/Research Support|Spero Therapeutics: Grant/Research Support|Suzhou Sinovent Pharmaceuticals Co.: Grant/Research Support|TauRx Therapeutics: Grant/Research Support|Tetraphase Pharmaceuticals: Grant/Research Support|tranScrip Partners: Grant/Research Support|Utility Therapeutics: Grant/Research Support|Valanbio Therapeutics, Inc.: Grant/Research Support|VenatoRx: Grant/Research Support|Wockhardt Bio AG: Grant/Research Support **Anthony P. Cammarata, M.S.**, Adagio Therapeutics, Inc: Grant/Research Support|Amplyx Pharmaceuticals, Inc.: Grant/Research Support|AN2 Therapeutics: Grant/Research Support|Antabio SAS: Grant/Research Support|Arcutis Biotherapeutics, Inc: Grant/Research Support|B. Braun Medical Inc.: Grant/Research Support|Basilea Pharmaceutica: Grant/Research Support|Boston Pharmaceuticals: Grant/Research Support|Celdara Medical LLC: Grant/Research Support|Cidara Therapeutics Inc: Grant/Research Support|Cipla USA: Grant/Research Support|Crestone Inc: Grant/Research Support|CXC: Grant/Research Support|Debiopharm International SA: Grant/Research Support|Entasis Therapeutics: Grant/Research Support|Evopoint Biosciences Co: Grant/Research Support|Fedora Pharmaceuticals: Grant/Research Support|GlaxoSmithKline: Grant/Research Support|Hoffmann-La Roche: Grant/Research Support|Insmed Inc.: Grant/Research Support|Iterum Therapeutics Limited: Grant/Research Support|Kaizen Bioscience, Co.: Grant/Research Support|KBP Biosciences USA: Grant/Research Support|Lassen Therapeutics: Grant/Research Support|Matinas Biopharma: Grant/Research Support|Meiji Seika Pharma Co., Ltd.: Grant/Research Support|Melinta Therapeutics: Grant/Research Support|Menarini Ricerche S.p.A.: Grant/Research Support|Mutabilis: Grant/Research Support|Nabriva Therapeutics AG: Grant/Research Support|Novartis Pharmaceuticals Corp.: Grant/Research Support|Paratek Pharmaceuticals, Inc.: Grant/Research Support|PureTech Health: Grant/Research Support|Sfunga Therapeutics: Grant/Research Support|Spero Therapeutics: Grant/Research Support|Suzhou Sinovent Pharmaceuticals Co.: Grant/Research Support|TauRx Therapeutics: Grant/Research Support|Tetraphase Pharmaceuticals: Grant/Research Support|tranScrip Partners: Grant/Research Support|Utility Therapeutics: Grant/Research Support|Valanbio Therapeutics, Inc.: Grant/Research Support|VenatoRx: Grant/Research Support|Wockhardt Bio AG: Grant/Research Support **Kajal Larson, PhD**, Entasis Therapeutics: Employee **Kathryn Liolios, MA**, Adagio Therapeutics, Inc.: Grant/Research Support|Amplyx Pharmaceuticals, Inc.: Grant/Research Support|AN2 Therapeutics: Grant/Research Support|Antabio SAS: Grant/Research Support|Arcutis Biotherapeutics, Inc.: Grant/Research Support|B. Braun Medical Inc.: Grant/Research Support|Basilea Pharmaceutica: Grant/Research Support|Boston Pharmaceuticals: Grant/Research Support|Celdara Medical LLC: Grant/Research Support|Cidara Therapeutics Inc.: Grant/Research Support|Cipla USA: Grant/Research Support|Crestone Inc.: Grant/Research Support|CXC: Grant/Research Support|Debiopharm International SA: Grant/Research Support|Entasis Therapeutics: Grant/Research Support|Evopoint Biosciences Co.: Grant/Research Support|Fedora Pharmaceuticals: Grant/Research Support|GlaxoSmithKline: Grant/Research Support|Hoffmann-La Roche: Grant/Research Support|Insmed Inc.: Grant/Research Support|Iterum Therapeutics Limited: Grant/Research Support|Kaizen Bioscience, Co.: Grant/Research Support|KBP Biosciences USA: Grant/Research Support|Lassen Therapeutics: Grant/Research Support|Matinas Biopharma: Grant/Research Support|Meiji Seika Pharma Co., Ltd.: Grant/Research Support|Melinta Therapeutics: Grant/Research Support|Menarini Ricerche S.p.A.: Grant/Research Support|Mutabilis: Grant/Research Support|Nabriva Therapeutics AG: Grant/Research Support|Novartis Pharmaceuticals Corp.: Grant/Research Support|Paratek Pharmaceuticals, Inc.: Grant/Research Support|PureTech Health: Grant/Research Support|Sfunga Therapeutics: Grant/Research Support|Spero Therapeutics: Grant/Research Support|Suzhou Sinovent Pharmaceuticals Co.: Grant/Research Support|TauRx Therapeutics: Grant/Research Support|Tetraphase Pharmaceuticals: Grant/Research Support|tranScrip Partners: Grant/Research Support|Utility Therapeutics: Grant/Research Support|Valanbio Therapeutics, Inc.: Grant/Research Support|VenatoRx: Grant/Research Support|Wockhardt Bio AG: Grant/Research Support **Sarah McLeod, PhD**, Entasis Therapeutics: Employee **Alita Miller, PhD**, Entasis Therapeutics: Employee **Paul G. Ambrose, PharmD; MS; FIDSA**, Adagio Therapeutics, Inc.: Grant/Research Support|Amplyx Pharmaceuticals, Inc.: Grant/Research Support|AN2 Therapeutics: Grant/Research Support|Antabio SAS: Grant/Research Support|Arcutis Biotherapeutics, Inc.: Grant/Research Support|B. Braun Medical Inc.: Grant/Research Support|Basilea Pharmaceutica: Grant/Research Support|Boston Pharmaceuticals: Grant/Research Support|Bravos Biosciences: Ownership Interest|Celdara Medical LLC: Grant/Research Support|Cidara Therapeutics Inc.: Grant/Research Support|Cipla USA: Grant/Research Support|Crestone Inc.: Grant/Research Support|CXC: Grant/Research Support|Debiopharm International SA: Grant/Research Support|Entasis Therapeutics: Grant/Research Support|Evopoint Biosciences Co.: Grant/Research Support|Fedora Pharmaceuticals: Grant/Research Support|GlaxoSmithKline: Grant/Research Support|Hoffmann-La Roche: Grant/Research Support|ICPD: Ownership Interest|ICPD Biosciences, LLC.: Ownership Interest|ICPD Holdings: Ownership Interest|ICPD Technologies: Ownership Interest|Insmed Inc.: Grant/Research Support|Iterum Therapeutics Limited: Grant/Research Support|Kaizen Bioscience, Co.: Grant/Research Support|Matinas Biopharma: Grant/Research Support|Meiji Seika Pharma Co., Ltd.: Grant/Research Support|Melinta Therapeutics: Grant/Research Support|Menarini Ricerche S.p.A.: Grant/Research Support|Mutabilis: Grant/Research Support|Nabriva Therapeutics AG: Grant/Research Support|Novartis Pharmaceuticals Corp.: Grant/Research Support|Paratek Pharmaceuticals, Inc.: Grant/Research Support|PureTech Health: Grant/Research Support|Sfunga Therapeutics: Grant/Research Support|Spero Therapeutics: Grant/Research Support|Suzhou Sinovent Pharmaceuticals Co.: Grant/Research Support|TauRx Therapeutics: Grant/Research Support|Tetraphase Pharmaceuticals: Grant/Research Support|tranScrip Partners: Grant/Research Support|Utility Therapeutics: Grant/Research Support|Valanbio Therapeutics, Inc.: Grant/Research Support|VenatoRx: Grant/Research Support|Wockhardt Bio AG: Grant/Research Support **Ruben Tommasi, PhD**, Entasis Therapeutics: Employee **John O'Donnell, B.S**, Entasis Therapeutics: Employee

**Figure:** Abbreviations: %T>MIC, percentage of time on Day 1 that concentration exceed minimum inhibitory concentration; AUC, area under the concentration-time curve; CLcr, creatinine clearance; ELF, epithelial lining fluid; MIC, minimum inhibitory concentration; q6h, every 6 hours; PK-PD, pharmacokinetic/pharmacodynamic; PTA, probability of PK/PD target attainment

